# Supplementary material for: Microglia innately develop within cerebral organoids
Source: Nat Commun. 2018 Oct 9;9:4167. doi: 10.1038/s41467-018-06684-2 (PMC6177485; doi:10.1038/s41467-018-06684-2)
Supplement: Supplementary file 1 — Supplementary Information [file 41467_2018_6684_MOESM1_ESM.pdf]

# Supplementary Information "Microglia Innately develop within cerebral organoids"

Ormel et al.

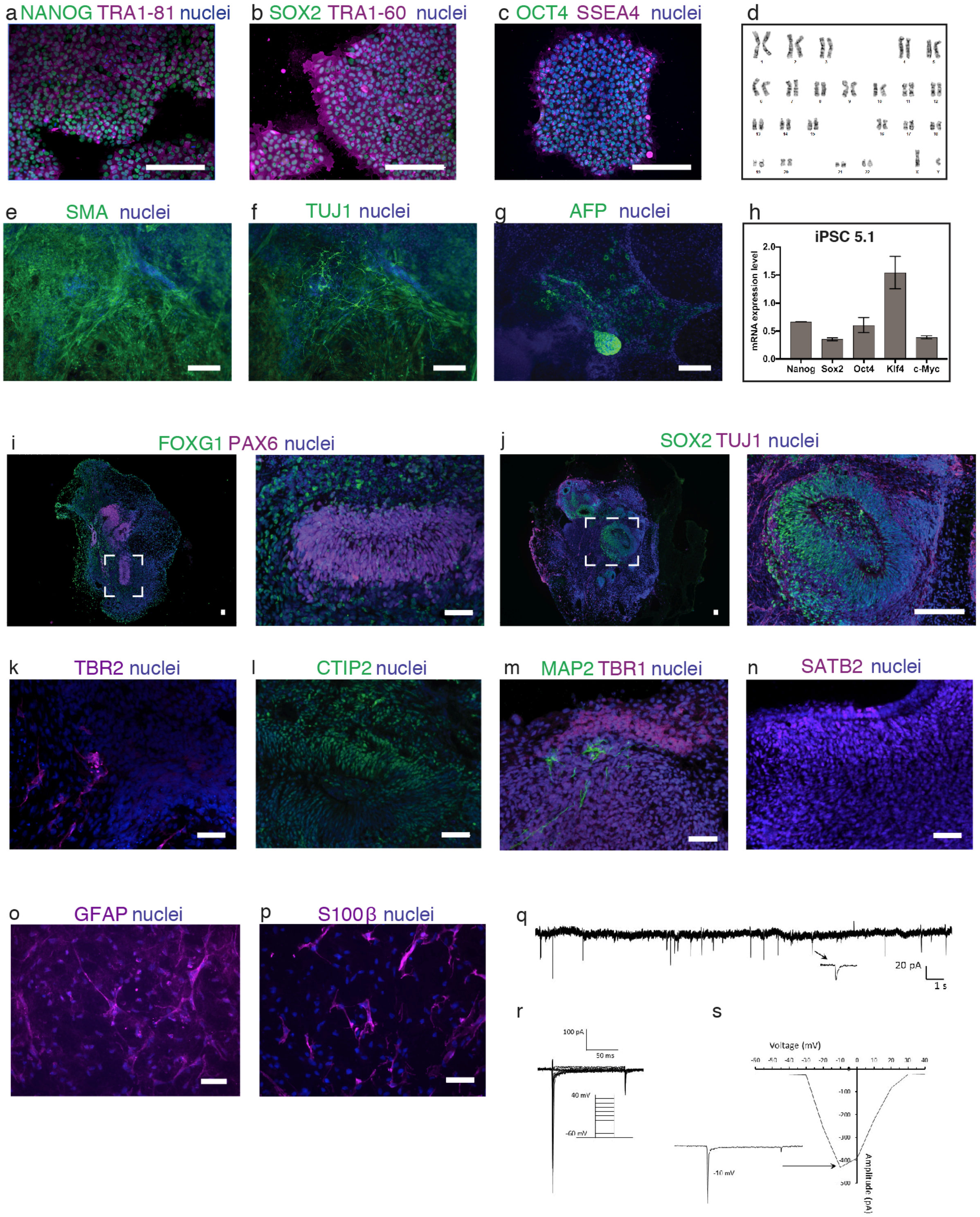


**Supplementary Figure 1.** iPSC5 characterization and neuronal identity in organoids

a to c- Stem cell markers NANOG and TRA1-81 (a), SOX2 and TRA1-60 (b), and OCT4 and SSEA4 (c) expression in iPSC5 line. Scale bar 200 $\mu$m.

d- Karyogram of iPSC 5.

e to g- Pluripotency potential was evaluated by spontaneous differentiation assay followed by immunohistochemistry for smooth muscle actin (SMA, e), β-III Tubulin (TUJ1, f), and α-fetoprotein (AFP, g). Scale bar 200 μm.

h- mRNA expression of stem cell markers by qRT-PCR (normalized to *ACTB*) with a commercial embryonic stem cell line (HUES6). mRNA expression of iPSC 5 relative to HUES6.

i- Dorsal forebrain identity of organoids assessed by FOXG1 and PAX6 immunostainings at day 31. Representative pictures of organoids from iPSC 1 are shown. (right panel is a close-up of the left panel) Scale bars 200μm.

j- Neuronal features of the organoids was evaluated by immunohistochemistry for progenitor marker SOX2 and pan-neuronal marker TUJ1 at day 31. Representative pictures of organoids derived from iPSC 1 are shown. (right panel is a close-up of the left panel) Scale bars 200 μm.

k, l and m- Presence of TBR2$^+$ progenitors (k), cortical deep layer neurons expressing CTIP2 (l) and TBR1 (m), and mature neurons MAP2 (m) in cerebral organoids at day 31. Representative pictures of organoids from iPSC 1 are shown. Scale bars 200 μm.

n, o and p- Superficial layers were assessed by SATB2 expression at day 31 (n) and presence of astrocytes evaluated by expression of GFAP (o) and S100β (p) at day 52. Representative pictures of cerebral organoids from iPSC 1 are shown. Scale bars 200 μm.

q, r, s- Glutamatergic spontaneous excitatory postsynaptic potentials (sEPSCs) detected at a holding potential of -65 mV. Voltage dependent sodium currents, necessary for the generations of spikes, could be elicited (q). Example of a single sodium current evoked at -10 mV is shown (r). IV plot depicting the typical course of the voltage dependency of the sodium currents is shown (s). Cerebral organoids from iPSC 1 were used for the electrophysiology.

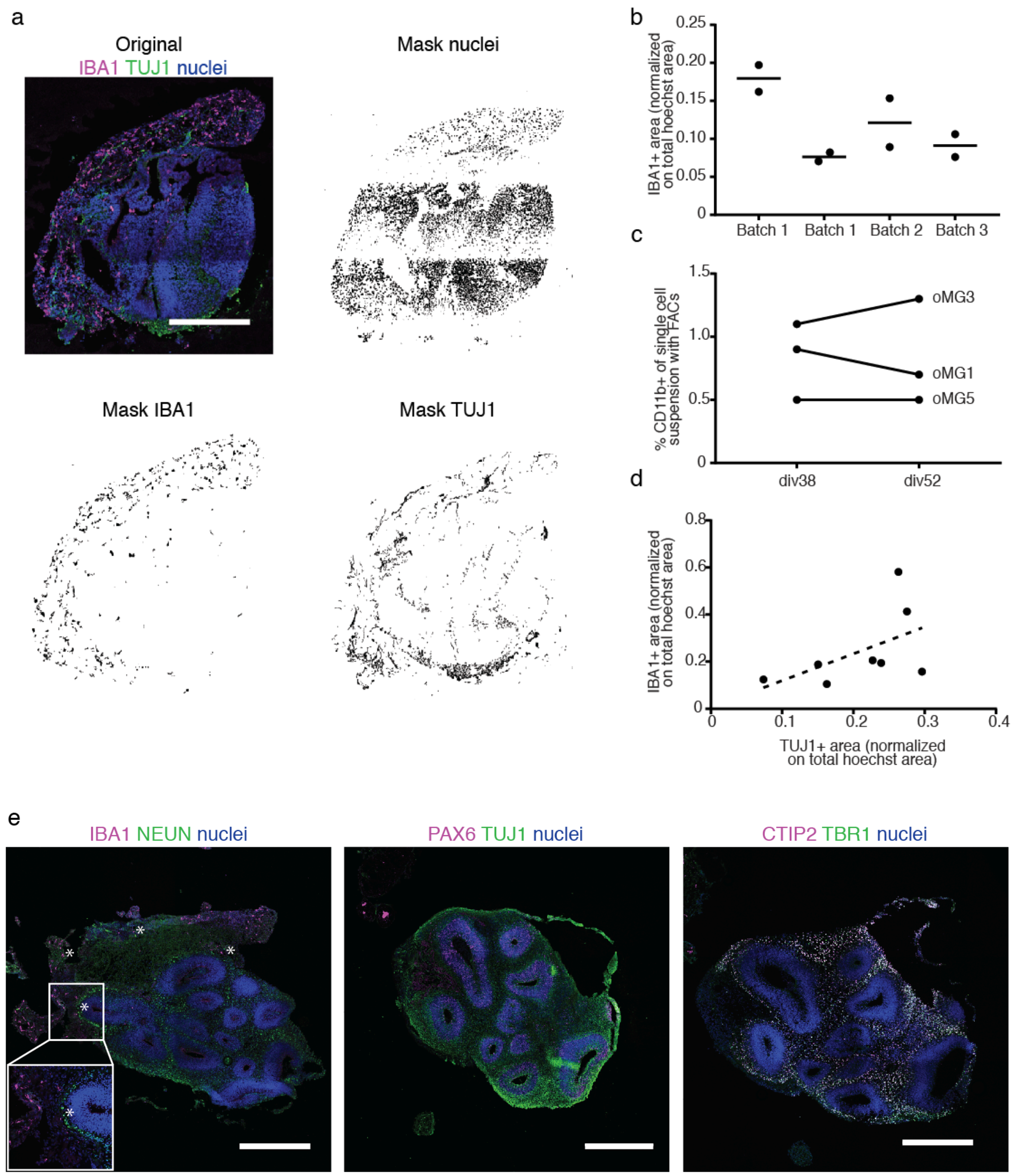


**Supplementary Figure 2.** The quantity of microglia is similar between batches, donors, and timepoints in culture and co-mature with neurons

a- Mask images created by an automated macro in FIJI to quantify the fraction of nuclei, IBA-1, and TUJ1 positive area of a tiled image of a fluorescent staining. The fluorescent channels were split and a separate threshold was applied to enable further analyses. Representative pictures of cerebral organoids from iPSC 1 are shown. Scale bar 500 $\mu$m.

b- Quantification of IBA-1/nuclei ratio from tiled fluorescent images of 2 sections per organoid. The variation between batches is similar to the variation between organoids of the same batch ranging from 0.05-0.2.

c- Percentage of $CD11b^+$ cells in organoid single cell suspension when sorted with flow cytometry. Organoids from three donors were used for this experiment (iPSC 1, 3, and 5) at two timepoints (38 and 52 days *in vitro*). The mean percentage of $CD11b^+$/$CD45^+$ cells of donors iPSC 1, 3, and 5 was 0.83% ± 0.3 (SD) at both time points for oMG (n = 6)

d- The increase of $IBA1^+$ is positively correlated with TUJ1. Each data-point reflects the IBA-1 and TUJ1 fraction, normalized to nuclei, of one tiled image of organoids from three separate batches.

e- Neuronal identity and cyto-architecture is maintained in organoids containing microglia as shown in tiled pictures from sections of one organoid after 66 days in culture. Co-staining for: microglia (IBA-1) and mature neurons (NEUN) (* indicates $NEUN^+$ cells in close proximity with $IBA\text{-}1^+$ cells, left panel); radial glia (PAX6) and a pan-neuronal marker (TUJ1, middle panel); and for a deep cortical layer marker (CTIP2) and a post-mitotic projection neuron marker (TBR1, right panel). Scale bar 500 $\mu$m.

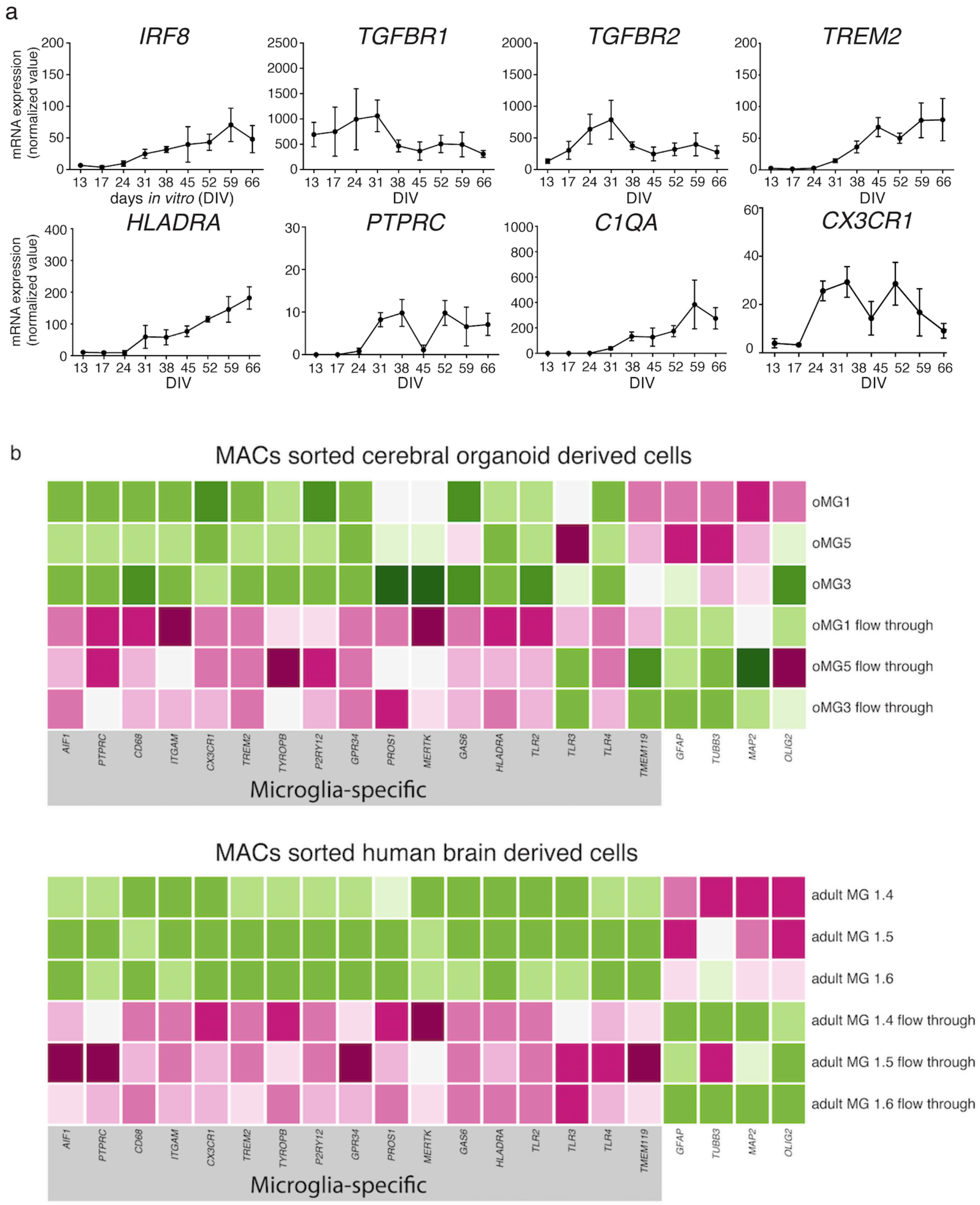


**Supplementary Figure 3**. oMG express microglia-specific genes that could be measured in the whole organoid but also when the microglia population is enriched

a- Graphs depicting mRNA expression levels of microglia-specific genes in organoids. mRNA levels were determined by qRT-PCR and normalized to the geomean of the reference genes *SDHA2* and *ACTB*. Data represent the mean of four batches consisting of two

organoids per batch per time-point. All batches consisted of organoids derived from iPSC 1. Error bars represent the standard error of the mean (SEM).

b- Magnetic automated cell sorting validation in oMG (upper panel) and adult MG (lower panel) by qRT-PCR by using a panel of classical microglia genes but also genes that should not be expressed by microglia (*GFAP, TUBB3, MAP2*, and *OLIG2*). mRNA levels were compared with the flow through fraction (oMG flow through and adult MG flow through). Data was log transformed and scaled for each sample to visualize the expression pattern. mRNA levels were determined by qRT-PCR and normalized to the geomean of the reference genes *SDHA2* and *ACTB*. n = 3 separate experiments in which oMG were isolated with $CD11b^+$ magnetic cell sorting from 8 organoids per experiment and also an n = 3 separate experiments to enrich adult MG with $CD11b^+$ magnetic cell sorting from fresh human brain tissue. (*p < 0.05)

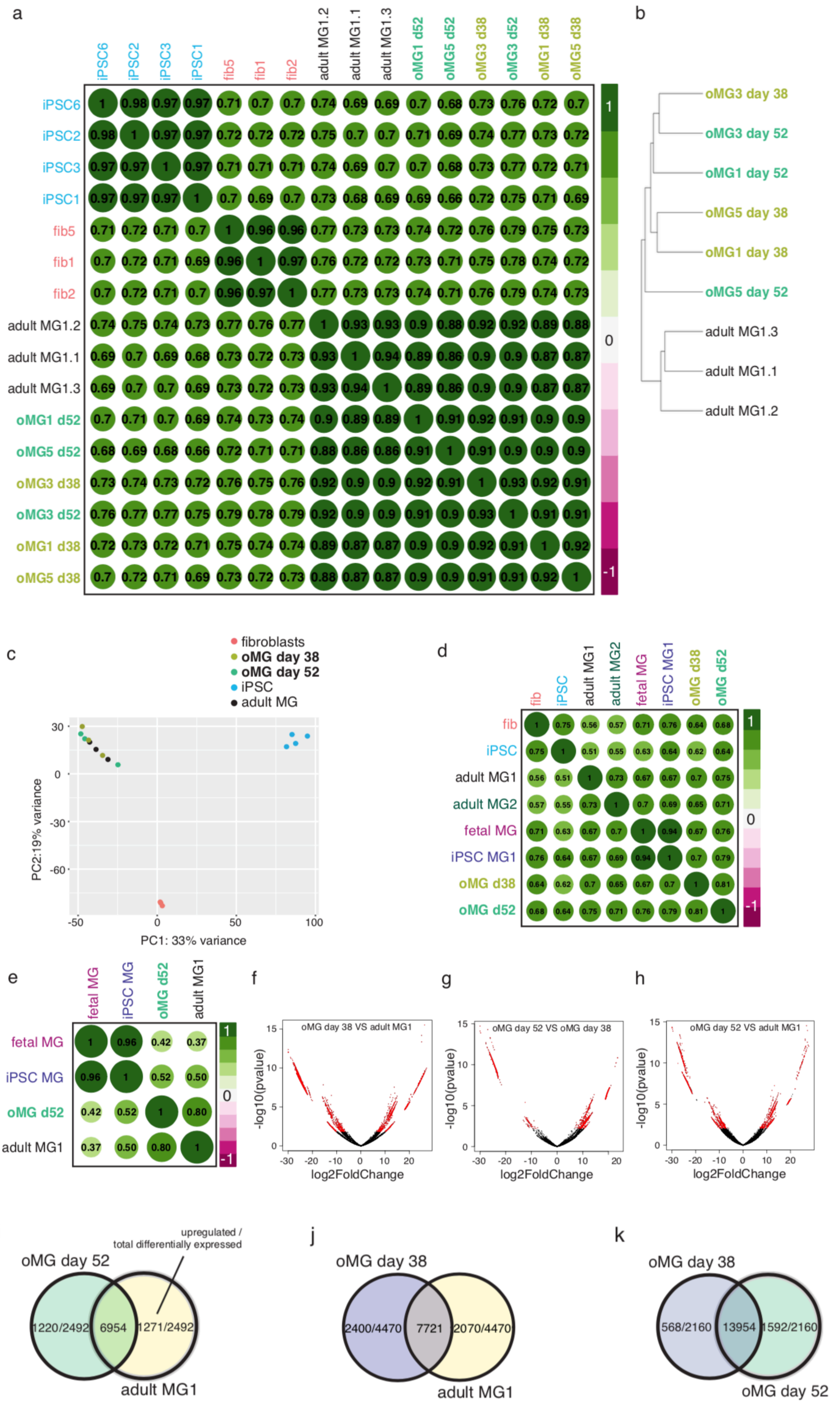
a
b
oMG3 day 38
oMG3 day 52
oMG1 day 52
oMG5 day 38
oMG1 day 38
oMG5 day 52
adult MG1.3
adult MG1.1
adult MG1.2
c
fibroblasts
oMG day 38
oMG day 52
iPSC
adult MG
PC1: 33% variance
PC2:19% variance
d
e
f
oMG day 38 VS adult MG1
g
oMG day 52 VS oMG day 38
h
oMG day 52 VS adult MG1
log2FoldChange
-log10(pvalue)
i
upregulated /
total differentially expressed
oMG day 52
1220/2492
6954
1271/2492
adult MG1
j
oMG day 38
2400/4470
7721
2070/4470
adult MG1
k
oMG day 38
568/2160
13954
1592/2160
oMG day 52

**Supplementary Figure 4**. Correlation among microglia-like cells

a- Spearman correlation analysis between oMG day 38, oMG day 52, adult MG 1, iPSC and fibroblast samples. DESeq2 rlog transformed raw gene counts of all genes annotated after removal of common genes (FDR > 0.05, sum of raw read counts > 0) between the samples were used as input. Size and color of circles indicate the strength and direction of the correlation, respectively.

b- Unsupervised hierarchical cluster analysis on DESeq2 rlog transformed raw counts of oMG day 38, oMG day 52, and adult MG1 based on all genes after removal of common genes (FDR > 0.05, sum of raw read counts > 0) between samples.

c- Principal component analysis on DESeq2 rlog transformed raw counts of oMG day 38, 52, adult MG, iPSC and fibroblasts.

d- Spearman correlation analysis between oMG day 38, 52, adult MG1, fetal MG, iPSC MG and adult MG2 scaled log transformed FPKM values of genes used in figure 3f. Median log transformed FPKM values for biological replicates were used as input for the correlation analysis.

e- Spearman correlation analysis between oMG day 52, adult MG, fetal MG, and iPSC MG scaled log2 FPKM values of a panel of transcription factor families that recognize microglia-related motifs. Median log transformed FPKM values for biological replicates were used as input for the correlation analysis.

f, g, and h- Volcano plots show differentially expressed genes (FDR < 0.05 in red) between day 38 oMG vs adult MG1 (f), day 52 vs day 38 oMG (g), and day 52 oMG vs adult MG1 (h).

i, j, and k- Venn diagrams show common expressed (FDR > 0.5, sum of raw read counts > 0) and differentially expressed genes (FDR < 0.05, enriched genes in sample/total amount of differentially expressed genes) between day 52 oMG vs adult MG1 (i), day 38 oMG vs adult

MG1 (j), and day 38 vs day 52 oMG (k) after shrinkage correction of the log2Fold change and removal of identified common genes between iPSC, fibroblasts, oMG and adult MG1.

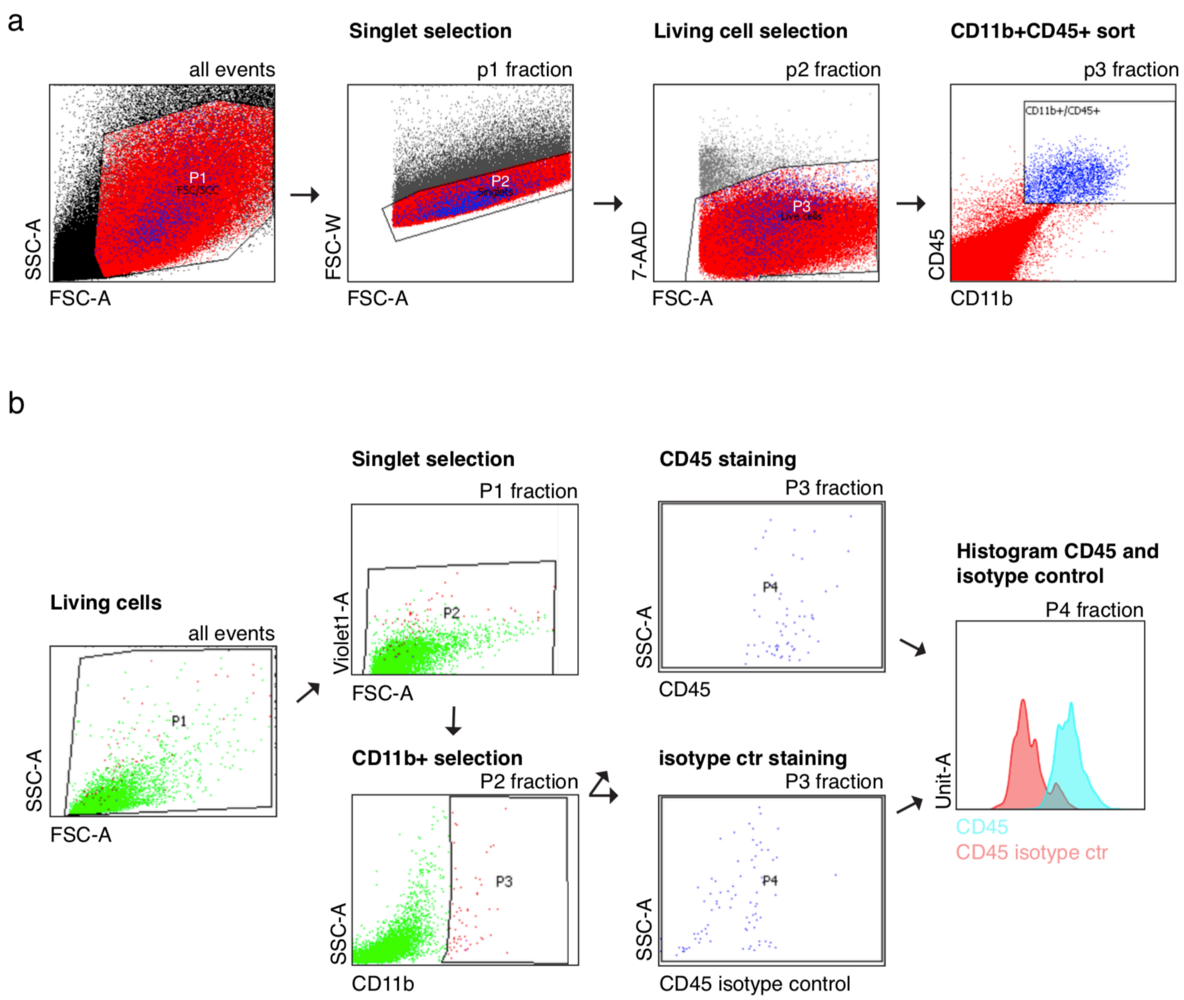


**Supplementary Figure 5**. Flow cytometry gating strategies used for cell sorting and protein expression quantification

a- Gating strategy to sort CD11b+CD45+ single living cells from the organoid/brain single cell suspensions. Representative gates of oMG enrichment of iPSC 3 are shown.

b- Gating strategy to quantify protein expression of CD11b+ cells gated from organoid/brain single cell suspension. Isotype controls of respective antibodies were used to accurately determine the protein expression. Representative gates of CD11b+ stained cells of iPSC 1 are shown.

Abbreviations: SSC-A = sideward scatter area; FSC-A = foreward scatter area; FSC-W = foreward scatter width.

**Supplementary Table 1.** Comparative overview of adaptations in the organoid differentiation protocol used in this study and the original protocol of Lancaster et al. 2014

| Description | | Ormel et al. 2018 | Lancaster et al. 2014 |
|---|---|---|---|
| Embryoid bodies generation | medium | hES4 (-P/S; +FBS); Y27 (1:100) | hES4 (-P/S; +FBS); Y27 (1:100) |
| | plates | AggreWell 800, (300 microwells/well) | V- bottom ULS 96 well plate |
| | Volume | 2 mL ($1.75x10^6$ cells per mL) | 150 µL ($6x10^4$ cells/mL) |
| | cells per embryoid body (EB) | Approx. 11500 cells | 9000 cells |
| Germ layer differentiation I | timing | up to day 4 | up to day 4 |
| | medium | hESC4 ; Y27 (1:100) | hESC4 ; Y27 (1:100) |
| | plates | flat bottom ULA 96 well plate | V- bottom ULA 96 well plate |
| | EB size | 320 (+/- 40) $\mu$m | > 350-400 $\mu$m |
| Germ layer differentiation II | timing | Days 4 to 6 | Days 4 to 6 |
| | medium | hES0 | hES0 |
| | plates | flat bottom ULA 96 well plate | V- bottom ULA 96 well plate |
| | EB size | 330 (+/- 37) $\mu$m | 350-600 $\mu$m |
| Induction of neural ectoderm | timing | days 6 to 12 | days 6 to 9 |
| | medium | NIM with 0.1 $\mu$g/mL Heparin | NIM with 1 $\mu$g/mL Heparin |
| | plates | flat bottom ULA 96 well plate | flat bottom ULA 24 well plate |
| | EB size | 330-570 $\mu$m | 500-600 $\mu$m |
| Transfer to Matrigel | timing | Day 13 | Day 11 |
| | medium | Differentiation medium without RA | Differentiation medium without RA |
| | plates | 60 mm petri dish | 60 mm tissue culture dish |
| Transfer bioreactor | timing | Day 17 | Day 15 |
| | medium | Differentiation medium with RA | Differentiation medium with RA |
| | platform | Spinning bioreactor | Spinning bioreactor |
| | speed | 27.5 rpm | 25 rpm |

**Supplementary Table 2.** Gene panels consisting of transcription factor genes important for microglia functioning *in vivo*

| Transcription family | Transcription factors important for microglia functioning *in vivo* |
|---|---|
| PU.1 | *SPI1* |
| CTCF | *CTCF* |
| IRF | *IRF1* |
| | *IRF2* |
| | *IRF3* |
| | *IRF8* |
| | *IRF9* |
| RUNX | *RUNX1* |
| | *RUNX2* |
| AP-1 | *JUN* |
| | *JUNB* |
| | *JUND* |
| | *FOS* |
| | *FOSB* |
| | *FOSL2* |
| | *ATF4* |
| | *BATF* |
| | *BATF2* |
| | *BATF3* |
| C/EBP | *CEBPA* |
| | *CEBPB* |
| | *CEBPG* |
| MEF2 | *MEF2A* |
| | *MEF2B* |
| | *MEF2C* |
| | *MEF2D* |
| SMAD | *SMAD3* |
| MAF | *MAF* |
| | *MAF1* |
| | *MAFB* |
| | *MAFF* |
| | *MAFG* |
| | *MAFK* |

**Supplementary Table 3.** Primer sequences used for qRT-PCR experiments

| Gene | 5'-Forward primer-3' | 5'-Reverse primer-3' |
|---|---|---|
| *ACTB* | GCTCCTCCTGAGCGCAAG | CATCTGCTGGAAGGTGGACA |
| *GAPDH* | TGTTCGACAGTCAGCCGCATCTTC | CAGAGTTAAAAGCAGCCCTGGTGA |
| *SOX2 endo* | CGAGGGAAATGGGAGGGGTGC | TGCAGCTGTCATTTGCTGTGGGT |
| *SOX2 viral* | GCATGACCAGCAGCCAGACCTA | TCTTGACCACGCTGCCCATGCT |
| *NANOG endo* | GCCTGTGATTTGTGGGCCTGA | GTGGAAGAATCAGGGCTGTCCTG |
| *OCT4 endo* | TGTCTCCGTCACCACTCTGGGC | CCCAAAAACCCTGGCACAAACTCC |
| *OCT4 viral* | AACCCCGAGGAAAGCCAGGACA | ACAGCACGCCCAGTGTCAGT |
| *C-MYC endo* | GCGGGCACTTTGCACTGGAACT | TTTCAGAGAAGCGGGTCCTGGCA |
| *C-MYC viral* | TACGCCCTGTTGAAGCTGGCTG | TGCACCGAGTCGTAGTCGAGGT |
| *C-MYC total* | ACCGAAAATGCACCAGCCCCA | CGATCTGGTCACGCAGGGCAAA |
| *KLF4 endo* | TCCCGCCGCTCCATTACCAA | TTTTGCCGCAGCCCGCGTAA |
| *KLF4 viral* | TGGAAGTTCGCCAGAAGCGACG | TTCATGTGCAGAGCCAGGTGGT |
| *dTOMATO* | TGAAGATGCGCGGCACCAACT | TGGTGGATCTCGCCCTTCAGCA |
| *SDHA2* | GAAGCCCTTTGAGGAGCACT | GTTTTGTCGATCACGGGTCT |
| *AIF1* | AGACGTTCAGCTACCCTGACTT | GGCCTGTTGGCTTTTCCTTTTCTC |
| *PTPRC* | GCAGCTAGCAAGTGGTTTGTTC | AAACAGCATGCGTCCTTTCTC |
| *CD68* | CTTCTCTCATTCCCCTATGGACA | GAAGGACACATTGTACTCCACC |
| *ITGAM* | TGCTTCCTGTTTGGATCCAACCTA | AGAAGGCAATGTCACTATCCTCTTGA |
| *CX3CR1* | CTTACGATGGCACCCAGTGA | CAAGGCAGTCCAGGAGAGTT |
| *TREM2* | TCAGGAAGGTCCTGGTGGA | GGGTGGGAAGGGGATTTCTC |
| *TYROPB* | TACGGCCTCTGTGTGTTGAG | CGGAAACAGCGTATCACTGAG |
| *P2RY12* | TTTGTGTGTCAAGTTACCTCCG | CTGGTGGTCTTCTGGTAGCG |
| *GPR34* | CCTGATGTCCAGTAACATTCGC | CATGCAGGGAGTATCCTGGT |
| *PROS1* | TTGCACTTGTAAACCAGGTTGG | CAGGAACAGTGGTAACTTCCAG |
| *MERTK* | CTCTGGCGTAGAGCTATCACT | AGGCTGGGTTGGTGAAAACA |
| *GAS6* | CTCTCTCTGTGGCACTGGTA | CCTTGATCTCCATTAGGGCCAA |
| *HLADRA* | CCCAGGGAAGACCACCTTT | CACCCTGCAGTCGTAAACGT |
| *TLR2* | ATCCTCCAATCAGGCTTCTCT | GGACAGGTCAAGGCTTTTTACA |
| *TLR3* | CAAACACAAGCATTCGGAATCTG | AAGGAATCGTTACCAACCACATT |
| *TLR4* | AGTTGATCTACCAAGCCTTGAGT | GCTGGTTGTCCCAAAATCACTTT |
| *TMEM119* | CTTCCTGGATGGGATAGTGGAC | GCACAGACGATGAACATCAGC |
| *GFAP* | AGGTCCATGTGGAGCTTGAC | GCCATTGCCTCATACTGCGT |
| *TUBB3* | GGCCTTTGGACATCTCTTC | CTCCGTGTAGTGACCCTTG |
| *MAP2* | CTCAGCACCGCTAACAGAGG | CATTGGCGCTTCGGACAAG |
| *OLIG2* | AGGACAAGAAGCAAATGACAG | TCCATGGCGATGTTGAGG |
| *IL6* | TGCAATAACCACCCCTGACC | TGCGCAGAATGAGATGAGTTG |
| *IL1B* | TTTGAGTCTGCCCAGTTCCC | TCAGTTATATCCTGGCCGCC |
| *CD163* | TTTGTCAACTTGAGTCCCTTCAC | TCCCGCTACACTTGTTTTCAC |
| *MRC1* | TGCAGAAGCAAACCAAACCTGTAA | CAGGCCTTAAGCCAACGAAACT |
| *TNF* | TGGAGAAGGGTGACCGACTC | TCACAGGGCAATGATCCCAA |
| *RUNX1* | AAGACCCTGCCCATCGCTTT | CATCATTGCCAGCCATCACAG |
| *SPI1* | GTGCAAAATGGAAGGGTTTCCC | TACTCGTGCGTTTGGCGTTG |
| *CSF1R* | ATCAGCATCCGGCTGAAAGT | CTCGAATCCGCACCAGCTCT |
| *IL34* | TGCACTGTCACGGGTTTTCT | CCCTCGTAAGGCACACTGAT |
| *CSF1* | GCAGGAGTATCACCGAGGAG | CACGAGGTCTCCATCTGACTG |
| *TGFB1* | CAATTCCTGGCGATACCTCAG | GCACAACTCCGTGACATCAA |
| *IRF8* | ATCAAAAGGAGCCCTTCCCC | ATCAAAAGGAGCCCTTCCCC |
| *TGFBR1* | TCCAAACCACAGAGTGGGAAC | TCCAAACCACAGAGTGGGAAC |
| *TGFBR2* | GTATCGCCAGCACGATCCCA | GAAACTTGACTGCACCGTTGTT |
| *C1QA* | GAGCACCAGACGGGAAGAAA | TAAGGCCTTGGATGCCTGTC |

**Supplementary Table 4.** Antibodies used in this study for immune histo/cytochemistry

| Antigen/target | Host species | Dilutions | Provider, article number |
|---|---|---|---|
| SMA | Mouse | 1:100 | Sigma, A2547 |
| AFP | Rabbit | 1:50 | Quartett, 2011200530 |
| Brachyury | Goat | 1:1000 | R&D systems, AF2085-SP |
| PAX6 | Mouse | 1:200 | DSHB, Pax6-s |
| NEUN | Mouse | 1:300 | Abcam, AB104224 |
| TUJ1 | Rabbit | 1:1000 | Sigma, T2200 |
| TUJ1 | Mouse | 1:1000 | Covance, MMS-435P |
| IBA-1 | Rabbit | 1:1000 | Wakko, 019-19741 |
| IBA-1 | Goat | 1:1000 | Abcam, AB5076 |
| FOXG1 | Rabbit | 1:100 | Abcam, AB18259 |
| CD68 | Rabbit | 1:100 | Invitrogen, MA5-13324 |
| CTIP2 | Rat | 1:100 | Abcam, AB18465 |
| TBR1 | Rabbit | 1:100 | Gift from Robert Hevner |
| S100β | Rabbit | 1:600 | Dako, Z0311 |
| GFAP-pan | Rabbit | 1:1000 | Dako, Z0334 |
| PSD-95 | Mouse | 1:300 | NeuroMab, 75028 |
| MAP2 | Mouse | 1:300 | Biolegend, SMI-52p |
| PU.1 | Rabbit | 1:100 | Invitrogen, A13971 |
| SATB2 | Rabbit | 1:300 | Abcam, AB34735 |
| NANOG | Rabbit | 1:200 | |
| OCT4 | Rabbit | 1:200 | |
| SSEA4 | Mouse | 1:200 | STEMLight iPSC characterization Kit, Cell Signalling, 9656S |
| TRA1-60 | Mouse | 1:200 | |
| TRA1-81 | Mouse | 1:200 | |
| SOX2 | Rabbit | 1:200 | |
| Mouse, 568 | Donkey | 1:1000 | ThermoFisher, A10037 |
| Mouse, 555 | Donkey | 1:1000 | ThermoFisher, A31570 |
| Mouse, 488 | Donkey | 1:1000 | ThermoFisher, A21202 |
| Rabbit, 568 | Donkey | 1:1000 | ThermoFisher, A10042 |
| Rabbit, 488 | Donkey | 1:1000 | ThermoFisher, A21206 |
| Goat, 488 | Donkey | 1:1000 | ThermoFisher, A11055 |
| Rat, 488 | Donkey | 1:1000 | ThermoFisher, A21208 |
| Rabbit, Atto647N | Goat | 1:200 | Sigma Aldrich, 40839 |
| Rabbit, 488 | Goat | 1:200 | ThermoFisher, A11034 |
| Mouse, 594 | Goat | 1:200 | ThermoFisher, A11032 |
