# Supplementary material for: Microglia innately develop within cerebral organoids
Source: Nat Commun. 2018 Oct 9;9:4167. doi: 10.1038/s41467-018-06684-2 (PMC6177485; doi:10.1038/s41467-018-06684-2)
Supplement: Supplementary file 5 — Description of Additional Supplementary Files [file 41467_2018_6684_MOESM5_ESM.pdf]

Description of Additional Supplementary Files

File Name: Supplementary Data 1
Description: Top 100 significant enriched genes in oMG day 52 vs adult MG1, adult MG1 vs oMG day 52, oMG day 38 vs day 52 and oMG day 52 vs day 38 (FDR < 0.05).

File Name: Supplementary Data 2
Description: Significant enriched Biological Processes GO terms (p-value < 0.05).
